# A molecular signature for the metabolic syndrome by urine metabolomics

**DOI:** 10.1186/s12933-021-01349-9

**Published:** 2021-07-28

**Authors:** Chiara Bruzzone, Rubén Gil-Redondo, Marisa Seco, Rocío Barragán, Laura de la Cruz, Claire Cannet, Hartmut Schäfer, Fang Fang, Tammo Diercks, Maider Bizkarguenaga, Beatriz González-Valle, Ana Laín, Arantza Sanz-Parra, Oscar Coltell, Ander López de Letona, Manfred Spraul, Shelly C. Lu, Elisabetta Buguianesi, Nieves Embade, Quentin M. Anstee, Dolores Corella, José M. Mato, Oscar Millet

**Affiliations:** 1 Precision Medicine and Metabolism Laboratory, CIC bioGUNE, BRTA, CIBERehd, Bizkaia Technology Park, Bld. 800, 48160 Derio, Bizkaia Spain; 2 OSARTEN Kooperativa Elkartea, 20500 Arrasate-Mondragón, Spain; 3 Department of Preventive Medicine and Public Health, School of Medicine, University of Valencia, 46010 Valencia, Spain; 4 CIBER Fisiopatología de la Obesidad y Nutrición, Madrid, Spain; 5 Bruker Biospin GmbH, Silberstreifen, 76287 Rheinstetten, Germany; 6 Department of Computer Languages and Systems, Universitat Jaume I, 12071 Castellón, Spain; 7 Getxo Kirolak, Los Chopos Etorbidea, 56, 48992 Getxo, Bizkaia Spain; 8 Karsh Division of Gastroenterology and Hepatology, Cedars-Sinai Medical Center, Los Angeles, CA USA; 9 Gastroenterology Department, University of Turin, Turin, Italy; 10 Translational & Clinical Research Institute, Faculty of Medical Sciences, Newcastle University, Newcastle upon Tyne, UK; 11 Newcastle NIHR Biomedical Research Centre, Newcastle Upon Tyne Hospitals NHS Trust, Newcastle upon Tyne, UK

**Keywords:** Metabolic syndrome, NMR spectroscopy, NMR-metabolomics, Precision medicine, Urine

## Abstract

**Background:**

Metabolic syndrome (MetS) is a multimorbid long-term condition without consensual medical definition and a diagnostic based on compatible symptomatology. Here we have investigated the molecular signature of MetS in urine.

**Methods:**

We used NMR-based metabolomics to investigate a European cohort including urine samples from 11,754 individuals (18–75 years old, 41% females), designed to populate all the intermediate conditions in MetS, from subjects without any risk factor up to individuals with developed MetS (4–5%, depending on the definition). A set of quantified metabolites were integrated from the urine spectra to obtain metabolic models (one for each definition), to discriminate between individuals with MetS.

**Results:**

MetS progression produces a continuous and monotonic variation of the urine metabolome, characterized by up- or down-regulation of the pertinent metabolites (17 in total, including glucose, lipids, aromatic amino acids, salicyluric acid, maltitol, trimethylamine *N*-oxide, and *p*-cresol sulfate) with some of the metabolites associated to MetS for the first time. This metabolic signature, based solely on information extracted from the urine spectrum, adds a molecular dimension to MetS definition and it was used to generate models that can identify subjects with MetS (AUROC values between 0.83 and 0.87). This signature is particularly suitable to add meaning to the conditions that are in the interface between healthy subjects and MetS patients. Aging and non-alcoholic fatty liver disease are also risk factors that may enhance MetS probability, but they do not directly interfere with the metabolic discrimination of the syndrome.

**Conclusions:**

Urine metabolomics, studied by NMR spectroscopy, unravelled a set of metabolites that concomitantly evolve with MetS progression, that were used to derive and validate a molecular definition of MetS and to discriminate the conditions that are in the interface between healthy individuals and the metabolic syndrome.

**Supplementary Information:**

The online version contains supplementary material available at 10.1186/s12933-021-01349-9.

## Background

Metabolic syndrome (MetS) is a complex disorder that puts together different health conditions. When untreated, MetS progressively leads to the development of metabolic abnormalities, elevates the risk for cardiovascular episodes and, ultimately, increases the mortality [1]. MetS constitutes a first order medical problem with a worldwide prevalence between 10 and 40% depending on the country or region [2]. This prevalence is directly attributed to unhealthy lifestyle habits, leading to a growing number of people affected by obesity or diabetes that are also associated with the development of MetS.

Albeit its importance, there is no consensus definition for MetS, in line with the complex nature of the syndrome. The current diagnostic of MetS is mostly based on the coincident identification of at least three from a set of known risk factors (RF, Table 1). Several relevant health institutions like the World Health Organization (WHO), the International Diabetes Federation (IDF), the National Cholesterol Education Program-Third Adult Treatment Panel (NCEP:ATP III), the European Group for the Study of Insulin Resistance (EGIR), and the American Association for Clinical Endocrinology (AACE) differ on which risk factors (RF) contribute and/or are essential for diagnosing MetS (bold-highlighted RFs in Table 1) [3–8]. There is consensus on some RF contributing to MetS: altered glucose metabolism, obesity, dyslipidemia and high blood pressure [9] but it is not clear how many of the contributing RF are required to diagnose MetS, nor the relation between a given combination of RF and the severity of the syndrome. In 2009, a seminal document attempted to unify some of the existing definitions for MetS and concluded that it emerges only when at least three of the abovementioned RF are present, with no single one being essential (Harmonized column in Table 1) [6]. Cut-off levels for each of the RF were also defined but this strategy suffers from the inherent difficulty to obtain a causal relationship between a RF and the syndrome.

**Table 1:** Definition criteria for the diagnosis of MetS according to the different organizations

	WHO	EGIR	AACE	NCEP:ATPIII	IDF	Harmonized
Glucose metabolism (FG MG/DL)	**IGT, IFG, T2DM or lowered insulin sensitivity**^**†**^	**IR**^**‡**^ FG ≥ 110	**IGT or IFG (but not diabetes)**^**†**^	FG ≥ 100	FG ≥ 100 or T2DM	FG ≥ 100 or treatment
Obesity (BMI KG/M^2^, WC CM)	WHR(m) > 0.90 WHR(f) ˃ 0.85 or BMI ˃ 30	WC(m) ≥ 94 WC(f) ≥ 80	BMI ˃ 25	WC(m) ≥ 102 WC(f) ≥ 88	**Elevated WC, ethnicity, and gender specific**^**†**^	Elevated WC, population, and country specific
Dyslipidemia (TG, HDL-C MG/DL)	TG ˃ 150 or HDL-C(m) ˂ 35 HDL-C(f) ˂ 39	TG ˃ 177 or HDL-C ˂ 39	TG ≥ 150 or HDL-C(m) ˂ 40, HDL-C(f) ˂ 50	TG ≥ 150 or HDL-C(m) ˂ 40, HDL-C(f) ˂ 50	TG ≥ 150 or treatment or HDL-C(m) ˂ 40, HDL-C(f) ˂ 50 or treatment	TG ≥ 150 or treatment or HDL-C(m) ˂ 40, HDL-C(f) ˂ 50 or treatment
Hypertension (BP MMHG)	≥ 140/90	≥ 140/90	≥ 130/85	≥ 130/85	≥ 130/85 or treatment	≥ 130/85 or treatment
Other factors	Microalbominuria ˃ 30 mg/g	Not relevant	Other risk factors^§^	Not relevant	Not relevant	Not relevant

Organizations: WHO: World Health Organization; EGIR: European Group for the Study of Insulin; AACE: American Association of Clinical Endocrinology; NCEP:ATPIII: National Cholesterol Education Program-Third Adult Treatment Panel; IDF: International Diabetes Federation

IFG: impaired fasting glucose; IGT: impaired glucose tolerance; FG: fasting plasma glucose; T2DM: type 2 diabetes; WC: waist circumference; WHR: waist-hip ratio; BMI: body mass index; TG: triglycerides; HDL-C: HDL cholesterol; BP: blood pressure; m: male; f: female

^†^Bold highlighted factors are compulsory for the given definition. Obtained from refs. [6, 27, 46]

^‡^IR: Insulin resistance, defined as hyperinsulinemia: top 25% of fasting insulin values among the nondiabetics

^§^Family history of T2DM, sedentary lifestyle, advanced age, ethnic groups susceptible to T2DM, polycystic ovary syndrome

Another unresolved issue is the putative relationship between MetS and non-alcoholic fatty liver disease (NAFLD), which is commonly considered to be the hepatic manifestation of the metabolic syndrome [10], mostly due to their congruent RF. Yet, there is little experimental evidence linking both diseases, and whether NAFLD and MetS are different expressions of the same disease or related comorbidities remains an open question.

All these ambiguities underline the need for new more objective and accurate signatures of MetS, ideally based on molecular and quantifiable descriptors. Metabolomics is a powerful tool to investigate MetS since all its contributing RF are expected to significantly alter metabolism [11]. Urine is metabolically very concentrated, not homeostatized and the very large number of metabolites found in urine may properly account for all the contributing RF to MetS [12–15]. In turn, NMR is particularly adequate for the analysis of complex solutions such as plasma, serum and urine [16] and it has been applied to study MetS, in serum samples so far [17].

In here, we have investigated MetS by using a large cohort of individuals mostly from a Southern European population (two Spanish regions), analysing close to 12,000 urine samples by NMR spectroscopy. The cohort includes volunteers of the general population and patients that presented one or several RF associated to MetS. An integrative analysis of this large spectra database allowed corroborating some of the already reported biomarkers, reporting novel ones and, most importantly, obtaining a metabolic signature of MetS progression and identifying the relative contributing risk for each factor.

## Methods

### Sample cohorts from healthy individuals and patients

A large cohort including individuals (n approx. 12,000) with different degree of the MetS was collected from this specific study. This cohort consisted of four different subcohorts (OSARTEN, OBENUTIC, PREDIMED and KIROLGETXO) recruited in a European country (Spain) and another one in different European regions (NAFLD). The relevant data for each subcohort is summarized in the Supplementary material (text and Additional file 1: Tables S1–S5). The procedures for sample collection and handling were the same one for every subcohort under consideration and abided standard operating procedures. Following the Declaration of Helsinki principles, all participants in the study provided informed consent to clinical investigations, with evaluation and approval from the corresponding ethics committee. All data was anonymized to protect the confidentiality of participants.

### Sample preparation

Samples were stored at − 80 °C and, on the day of the analysis, were defrosted at room temperature during 30 min. Aliquots were centrifuged at 6000 rpm for 5 min at 4 °C and then 630 μL of the supernatant were transferred into a 1.5 mL tube. Subsequently, 70 μL of a phosphate buffer (1.5 M KH_2_PO_4_/K_2_HPO_4_, 2 mM NaN_3_, 1% TSP in 70% D_2_O, pH 7.4) were added in the same microcentrifuge tube to minimize pH variation. The mix of urine and buffer was briefly vortexed and 600 μL of the mixture were finally transferred into a 5 mm NMR tube.

### NMR measurements

Experiments were performed as previously described [18, 19]. In brief, two complementary experiments were recorded per sample: a one-dimensional (1D) ^1^H spectrum with water presaturation for metabolite quantification and a two-dimensional (2D) J-resolved ^1^H spectrum. For selected samples, a 2D ^1^H,^1^H- TOCSY (TOtal Correlation SpectroscopY) spectrum was also recorded to confirm metabolite identification. Metabolites were identified from the 1D ^1^H NMR spectra using the Chenomx NMR software (version 8.6) and corroborated by experimental spiking when necessary.

### Filtering of samples

A multivariate clustering algorithm, DBSCAN (Density-based spatial clustering of applications with noise), was used with bins as input variables after Pareto scaling. After filtering and validation of the general characteristics, a total of 9,367 (94%), 960 (98%), 465 (96%), 246 (100%) and 101 (100%) of the samples for the OSARTEN, PREDIMED, OBENUTIC, NAFLD and KIROLGETXO subcohorts were further considered as valid samples.

### Statistical analysis

A cohort composed of OSARTEN, OBENUTIC, and PREDIMED subcohorts was used to analyse the 16 pathological conditions. A principal component analysis (PCA) was used to summarize and visualize (by PC 1 and 2) each condition, which was represented by its average profile. Each pathological condition was compared with the apparently healthy (0000) one. This comparison employed Wilcoxon nonparametric hypothesis testing for each bin to identify those with a statistically significant difference (p-value < 0.05), after adjustment by the False Discovery Rate (FDR) method to control for Type I errors due to multiple comparisons. Binary logarithms of fold-changes (log_2_FC) were used to quantify the magnitude and direction of differences. Fold-changes were calculated as the average of a variable within the target condition divided by its average within the apparently healthy condition.

Different conditions and bins were clustered and organized as dendrograms in heatmaps, using hierarchical clustering by the complete-linkage method and Euclidean distances. To quantify differences between average profiles of conditions, a multivariate Euclidean distance (with autoscale) was calculated between the apparently healthy and all other conditions. Resulting distances were scaled (range 0 to 1) and translated into a colour code for a graph connecting the different adjacent conditions, which was generated with *igraph* (R package version 1.2.6).

### Classification models for MetS

For each available MetS definition a binary classification model was built, with heatmap selected bins as input and MetS diagnosis (no/yes) as output. The data was randomly divided into training (75%) and testing (25%) sets. The performance was summarized in ROC curves for each MetS definition, including their AUCs with pertaining 95% confidence intervals and cut-off points to maximize the Youden index with associated specificity and sensitivity parameters.

### Microalbuminuria analysis

A semi-quantitative analysis using a test strip was done to each urine sample for the detection of proteinuria. The output results were considered as negative/positive if the value of proteinuria (identified as microalbuminuria) was lower/higher than 10 mg/dL.

## Results

### Setting the problem

To investigate the molecular signature of MetS, we first identified the RF that may contribute to the syndrome from the general characteristics of the donors. Four factors have well-known association with the development of MetS and they have been included in this study (Table 1): alterations in glucose metabolism, obesity (determined from BMI since waist circumference was inaccessible), dyslipidemia and hypertension. The WHO also considers microalbuminuria as a potential RF, but it is not routinely determined in all the medical check-ups and we have evaluated its putative influence in MetS with an independent sub-study (vide infra).

Our study was designed not only to investigate the contribution for each of the RF to MetS independently, but also to evaluate all their possible combinations, a total of 16 (2^4^) different conditions. We used a nomenclature for the conditions where the digits represent the four risk factors (RF_1_ RF_2_ RF_3_ RF_4_), binary coded by "1" or "0" to indicate that the given factor is present or absent in the condition (Table 2). According to this notation, a 0000 sample would originate from an apparently healthy subject while, for instance, a sample encoded as 1011 would belong to a patient that has diabetes, dyslipidemia and hypertension, but no obesity. A quantitative definition for the inclusion criteria for each of the RF is also listed in Table 2.

**Table 2:** Risk factors and conditions under consideration in this study

Conditions* (RF_1_, RF_2_, RF_3_, RF_4_)			
RF_1_ (Pre)Diabetes	RF_2_ Obesity	RF_3_ Dyslipidemia	RF_4_ Hypertension
Fasting plasma glucose > 100 mg/dL Previously diagnosed type 2 Diabetes, impaired fasting glucose, impaired glucose tolerance or insulin resistance taking medication for hyperglycemia	BMI ˃ 30 kg/m^2^	Triglycerides > 150 mg/dL HDL Cholesterol < 34.75 mg/dL in men or < 38.61 in women Previously diagnosed hypercholesterolemia, hyperlipidemia or hypertriglyceridemia taking medication for dyslipidemia	Blood pressure ≥ 140/90 mmHg Previously diagnosed hypertension taking medication for hypertension

**0/1** = absence/presence of the risk factor			
0000	Apparently healthy	1001	Diabetes + hypertension
0001	Hypertension	1010	Diabetes + dyslipidemia
0010	Dyslipidemia	1100	Diabetes + obesity
0100	Obesity	0111	Obesity + dyslipidemia + hypertension
1000	Diabetes	1011	Diabetes + dyslipidemia + hypertension
0011	Dyslipidemia + hypertension	1101	Diabetes + obesity + hypertension
0101	Obesity + hypertension	1110	Diabetes + obesity + dyslipidemia
0110	Obesity + dyslipidemia	1111	Diabetes + obesity + dyslipidemia + hypertension

RF: risk factor

Additional file 1: Table S6 shows the number of samples allocated to each condition (including OSARTEN, PREDIMED and OBENUTIC subcohorts), also stratified by sex. The apparently healthy condition is more prevalent than the rest of conditions, due to the characteristics of the OSARTEN subcohort, formed of active population. Even though some conditions are less prevalent, the number of samples in each condition is enough to reach high statistical power. In the worst case (1110, with 62 samples), it is still possible to detect a Cohen's small-medium effect size with more than 80% power in comparisons with the apparently healthy condition.

### The urine ^1^H NMR spectrum is sensitive to MetS

**Fig. 1:**
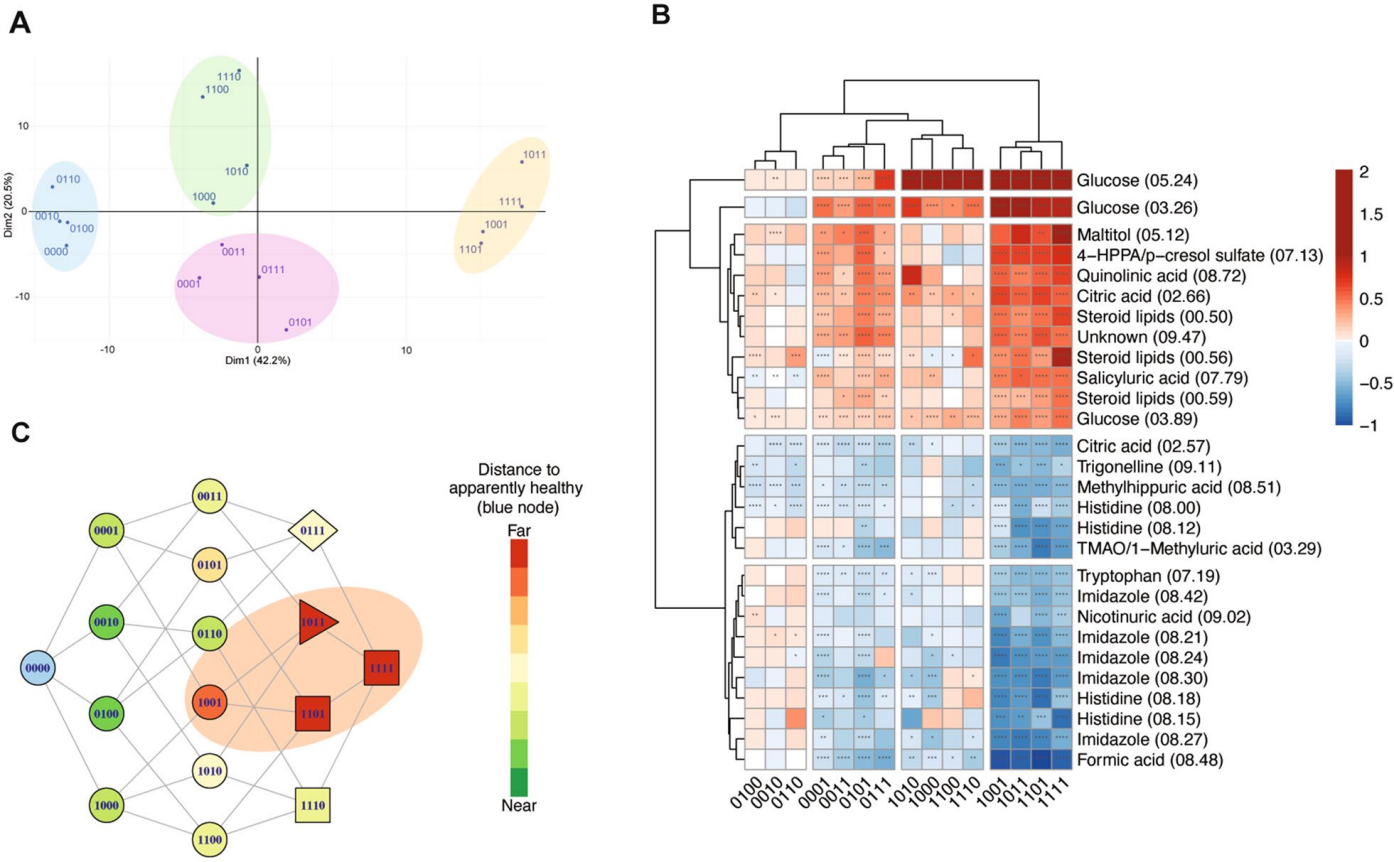
Univariate and Multivariate analyses for the MetS subtypes. **A** PCA for the mean profiles for the 16 conditions under consideration. Each condition contains (or not) the risk factor according to Table 1. Color ellipses indicates clusters for subjects with: diabetes (green), hypertension (purple), both factors (yellow) or none of the two (blue). **B** Heatmap for the different conditions as compared to the apparently healthy condition (0000). The conditions (in the abscise axis) and the bins/metabolites (in the ordinate axis) have been sorted according to cluster analysis. The relevant bins that contributed to the heatmap have been assigned to the corresponding metabolite, as indicated. The fold change is colour-coded according to the bar legend. For each condition, the statistical significance of the variation with respect to apparently healthy individuals is determined by the p-value, shown inside the squares. **C** Spearman correlation distances to the healthy condition for all the conditions. Colours represent the distance to the apparently healthy (0000) condition, as indicated in the legend. The lines connect adjacent conditions. MetS definition according to WHO, EGIR and AACE is represented by squares and triangles; definition from NCEP:ATPIII and Harmonized is represented by squares, triangles and rhombus; definition by IDF is represented by squares and rhombus. 4-HPPA: 4-hydroxyphenylpyruvic acid; TMAO: trimethylamine N-oxide. The orange ellipse embraces all the conditions that would correspond to MetS according to our metabolic definition

NMR-based metabolomics of urine allows the quantification of several hundreds of metabolites that include central metabolism, xenobiotics, metabolites from microbiota and nutrition derivatives among others [20] and, therefore, is an optimal source of information for the metabolic characterization of MetS. An unsupervised PCA analysis of the urine NMR spectra of the different subcohorts (Additional file 1: Figure S1) reported no significant differences, validating their full inclusion in the study. From all classified spectra, an average spectrum was composed for each of the 16 conditions. A PCA analysis of their mean profiles (Fig. 1A) shows that all conditions separate well in 2D principal components space, highlighting a differential manifestation of RF in the urine spectrum. Interestingly, four well-differentiated clusters of conditions can be observed in the PCA plot, that always discriminates well between diabetes and hypertension (coloured ellipses in Fig. 1A), consistent with previous observations [15], while obesity and dyslipidemia are separated only within each cluster, indicating a lower level of modification of the urine metabolites induced by these two factors [21, 22].

Based on these results, we then compared each condition to the apparently healthy one (samples from individuals with 0000). The heatmap in Fig. 1B shows the results obtained from the univariate analysis of the acquired urine samples, considering the intensity of the spectral bins as variables. The conditions (in the abscise axis) and the bins/metabolites (in the ordinate axis) have been sorted according to unsupervised cluster analysis. The bins have been assigned to the contributing metabolites and up to 17 different metabolites (and one unassigned bin) contribute to the discrimination of the conditions (Table 3). For the metabolites that are present in more than one bin, the most significant bin was used for the metabolite quantification. For each condition, the p-value indicates the statistical significance of the variation with respect to apparently healthy individuals (see asterisks inside the squares), while the fold change is colour-coded according to the bar legend: a red/blue value in the heatmap indicates up/down regulation of the bin. In most cases, all the bins that correspond to a given metabolite produce consistent fold changes, while the small differences observed in the magnitude of the fold change can be attributed to the metabolic heterogeneity of certain bins. Yet, citric acid shows upregulation at the 2.66 ppm bin and downregulation at the 2.57 ppm bin (Fig. 1B). This is explained by the large sensitivity of citric acid to pH and osmolarity, that produces small changes in the chemical shift and the intensity of the (outer) bins vary accordingly (Additional file 1: Figure S2) [23].

**Table 3:** Summary of metabolites discriminating MetS

Metabolite^*^	Variable importance in the model	log_2_FC^†^	*P*-value^†^	Associated RF^‡^
Glucose	1056.86	1.66 (1.37, 1.94)	5.88e−97	RF_1_, this study and definition
Formic acid	436.74	− 0.79 (− 0.87, − 0.71)	3.53e−77	n. a
Steroid lipids	364.47	0.57 (0.3, 0.86)	3.68e−31	RF_3_, this study and definition
TMAO^§^/1-Methyluric acid	218.32	− 0.54 (− 0.7, − 0.38)	1.58e−30	RF_2_ [47]
Trigonelline	201.66	− 0.4 (− 0.5, − 0.3)	1.38e−06	RF_2_ [48]
Tryptophan	198.95	− 0.38 (-0.44, − 0.31)	1.38e−38	RF_2_ [48]
Quinolinic acid	192.41	0.41 (0.24, 0.59)	1.99e−17	RF_2_ [36]
Imidazole	184.05	− 0.57 (− 0.7, − 0.43)	1.20e−26	RF_4_ [29]
Histidine	181.71	− 0.56 (− 0.75, − 0.37)	8.42e−16	RF_4_ [29]
4-HPPA^§^/p-cresol sulfate	171.38	0.53 (0.4, 0.67)	1.56e−19	RF_1_ [43]
Salicyluric acid	164.22	0.42 (0.29, 0.56)	8.77e−14	RF_2_ [48]
Maltitol	155.43	0.65 (0.45, 0.85)	2.22e−05	RF_1_ [28]
Methylhippuric acid	153.23	− 0.45 (− 0.54, − 0.36)	3.79e−21	n.a
Nicotinuric acid	146.41	− 0.38 (− 0.5, − 0.27)	1.18e−09	RF_2_ [48]

n.a: not applicable

^*^For metabolites with more than one associated bin, those results with the higher abs(log2FC) are showed

^†^Binary logarithms of fold-changes (log2FC), their 95% confidence intervals and p-values were calculated between MetS and non-MetS conditions

^‡^Numbers in parentheses represent the bibliographic reference where this metabolite is related to the pertaining RF

^§^4-HPPA: 4-hydroxyphenylpyruvic acid; TMAO: trimethylamine *N*-oxide

Several important conclusions can be extracted from the heatmap: (i) MetS emerges as a complex metabolic scenario where some metabolites upregulate and some others are downregulated in urine, (ii) the (unsupervised) cluster analysis sorts the conditions in a way that naturally progresses towards the consensus definition of MetS (i. e., the conditions with more RF = 1 fall in the right side of the heatmap and vice versa); (iii) the metabolic variation is concomitant to the progression towards MetS, with close-to-linear variations of the metabolite concentrations as a function of the conditions; and (iv) most of the pertinent metabolites are related to the molecular pathophysiology of the RF under consideration (Table 3): aromatic amino acids and histidine have been already associated to MetS [24–26]; insulin resistance is obviously related with an increase in glucose [27] and/or with elevated urine levels of p-cresol sulfate [28]; hypertension is associated with low imidazole concentrations [29, 30]; upregulation of steroid lipids is a hallmark for dyslipidemia and obesity [31–34] and a set of the discovered metabolites are related to obesity [35], salicyluric acid [36] and trimethylamine N-oxide (TMAO) [37, 38]). In turn, we also associate here, for the first time, some other dysregulated metabolites to MetS: methylhippuric acid, maltitol, 4-hydroxyphenylpyruvic acid (4-HPPA), trigonelline, quinolinic acid and nicotinuric acid.

### Towards a molecular discrimination of MetS

To further illustrate the relationship between the observed metabolic changes and MetS, in Fig. 1C we sketched a correlation map where adjacent conditions differing by only one RF are connected by lines and coloured by their Spearman’s correlation distance to the apparently healthy condition (0000), as indicated. The graph shows once more that the variation of the urine metabolome (colors in Fig. 1C) agrees well with MetS progression (raising number of RF = 1). Furthermore, the graph also reveals that not all the factors equally contribute to MetS progression; instead, for a given number of accumulated RF, certain progression pathways are more pathogenic than others. This was used to generate a *molecular signature of MetS* (1111, 1101, 1011 & 1001; orange-highlighted in Fig. 1C), that partially differs from the MetS definitions, based on symptom accumulation. For instance, the conditions 1110 and 0111 are both considered as MetS by many definitions, but they would fall in an intermediate position between MetS and an apparently healthy metabotype, according to our analysis. On the other hand, condition 1001 (with just hypertension and diabetes) is metabolically closer to MetS despite being generally considered as non-MetS.

**Fig. 2:**
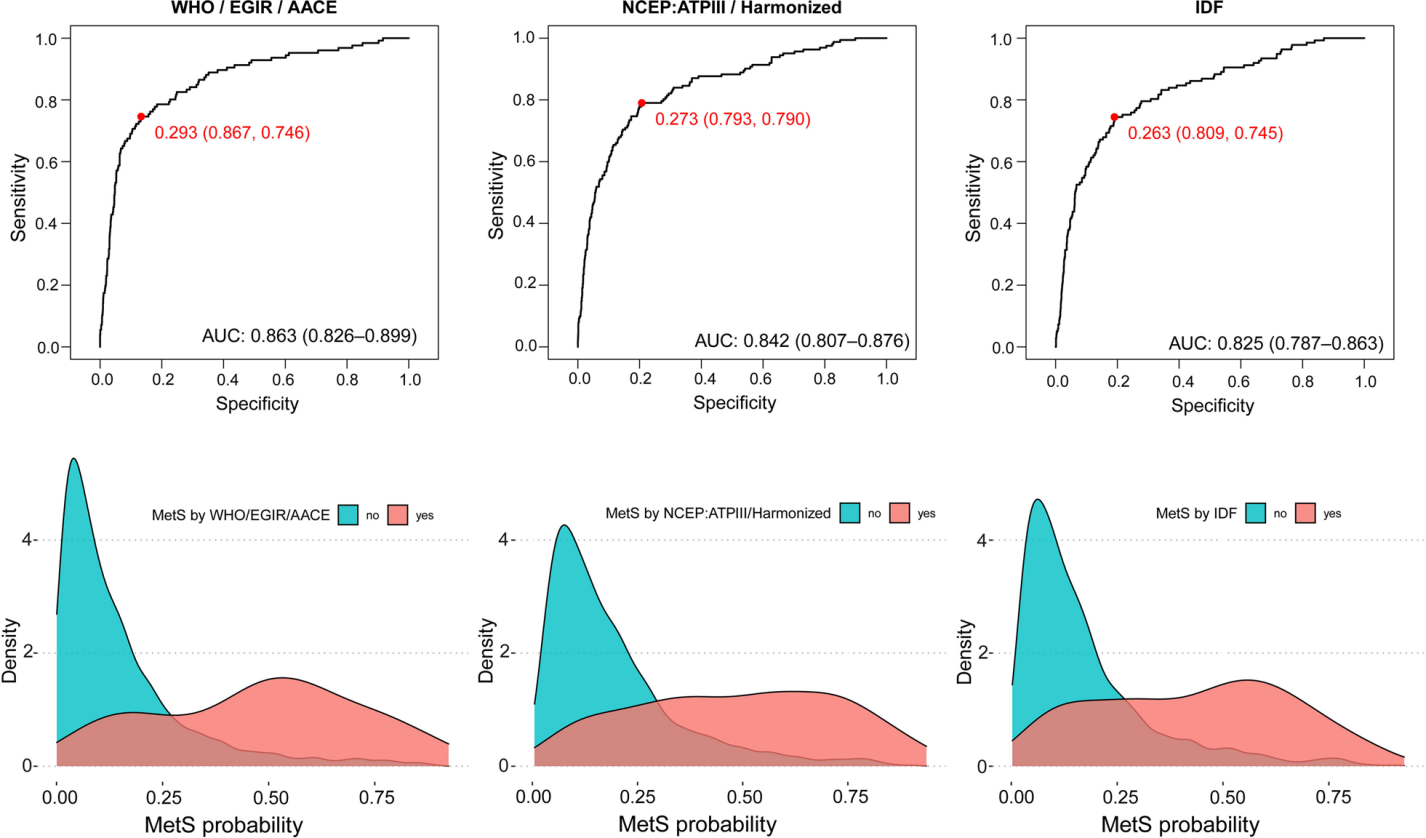
Probability distribution of the MetS models. **A**–**C** Receiving Operating Characteristic (ROC) curves for the three definitions under consideration: WHO, EGIR and AACE (A), NCEP:ATPIII and Harmonized (**B**), and IDF (**C**). **D**–**F** Smoothed histograms (kernel density based) showing the probability distributions of the MetS model applied to the full cohort for the three definitions under consideration: WHO, EGIR, and AACE (**D**), NCEP:ATPIII and Harmonized (**E**), and IDF (**F**). Red and green colours indicate that the sample has/doesn't have MetS according to the given definition, as indicated

We have also used the spectral database to create metabolic models of MetS (see Research Design and Methods for details) adapted to the different criteria used to define the MetS. To that end, we have first identified the number of cases with MetS condition, according to the different definitions, and using the general characteristics of the curated pool of 10,792 subjects. Only three out of the five different definitions from Table 1 can be truly distinguished with the general characteristics available in our cohort (here called independent definitions). Specifically, the MetS definition according to the WHO, EGIR and AACE are represented by the cluster of 1111, 1011, 1101 and 1110 conditions (squares and triangles in Fig. 1C); the MetS definition from NCEP:ATPIII and Harmonized are represented by the former conditions plus 0111 (squares, triangles and rhombus in Fig. 1C), and the IDF MetS definition is represented by the 1111, 1101, 1110 and 0111 conditions (squares and rhombus in Fig. 1C). Using these classifications, we found 642 cases for the NCEP:ATPIII or Harmonized definitions, 552 cases for the IDF definition and 494 cases for the WHO, EGIR or AACE definitions. Subsequently, we used the spectral information collected from the urine samples to train and test three metabolic models that maximizes the differences between the MetS and non-MetS conditions, one *per* independent definition and using 75% (8,094) /25% (2698) samples as training/validation cohorts. Figure 2A–C shows the ROC curves for the three models under consideration. Moreover, we have scrutinized the cohort, calculating its probability of undergoing MetS, for the three models/independent definitions (Fig. 2D–F). Specifically, after applying each model, samples were scored with a "MetS probability" between 0 and 1. The figure represents the distribution of these scores as a smoothed histogram (kernel densities). These plots evidence that people without MetS tend to cluster together in the region of low scores while people with MetS tend to be spread mainly along high score regions, also reflecting the heterogeneity of the syndrome. The results show that the models, based solely on the metabolomic analysis of urine samples, can identify MetS, in excellent compliance with all three independent definitions, with AUROC values between 0.83 and 0.87. We believe that the discrepancies reflect the differences between our molecular signature and the standard definitions for MetS. Indeed, while all independent definitions are largely consistent with our derived MetS metabotype, those including insulin resistance as mandatory criteria perform slightly better. This result is consistent with the statistical distance of the adiabetic 0111 condition that appears closer to the apparently healthy group than to full MetS (Fig. 1C). This condition is included in the NCEP:ATPIII, IDF and Harmonized definitions.

**Fig. 3:**
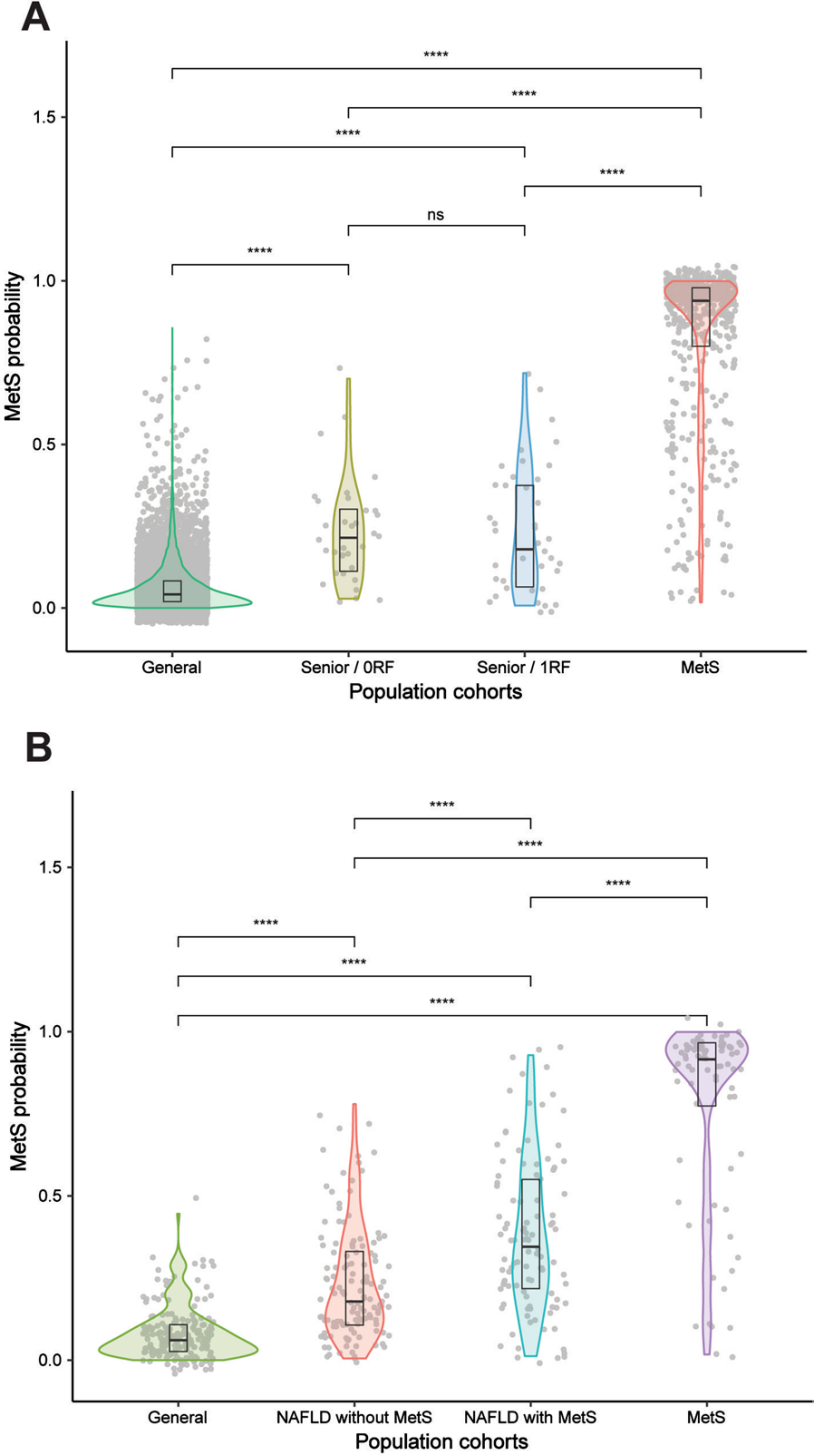
The effect of senior and NASH populations in MetS. **A** Probability distributions of suffering MetS calculated from the metabolic model for: general population (individuals with 0000, green), senior population with no risk factors (light green), senior population with 1RF (orange); population with MetS (blue). **B** Probability distributions of suffering MetS calculated from the metabolic model for: general population (individuals with 0000, green), MetS population (according to WHO definition, purple), NASH without MetS (orange), and NASH with MetS (blue)

Finally, the heatmaps segregated by gender (Additional file 1: Figure S3) renders equivalent results than the one obtained for the entire cohort (Fig. 1B), indicating that sex is not affecting the metabolic characterization of MetS. In turn, aging is a well-known risk factor for many diseases, including MetS [39]. The OSARTEN and OBENUTIC subcohorts are well-balanced in age while the PREDIMED cohort is older on average. A potential caveat is, therefore, that our metabolic model might partially monitor the aging process. To discard this pitfall, we also analysed an independent cohort (KIROLGETXO) that was not used in deriving our metabolic model and sampled a senior population (age between 60 and 85) with healthy lifestyle including regular sport activities. Not surprisingly, this cohort is enriched in people with none (n = 34) or only one MetS risk factor (n = 40) (Additional file 1: Table S4), and our metabolic model accordingly indicates only a very low probability for suffering MetS (Fig. 3A).

### The role of microalbuminuria and impaired renal function in MetS

As the WHO considers microalbuminuria as an RF for MetS, we also analysed the proteinuria values (> 10 mg/dL) for all the urine samples from the OSARTEN cohort. Since albumin is the main protein of the urine, we equated microalbuminuria with proteinuria. The OSARTEN cohort is large enough to represent most of the MetS conditions with sufficient statistical significance, despite being strongly biased towards the apparently healthy and more healthy conditions. Additional file 1: Figure S4 shows how the percentage of microalbuminuria increases as the condition approaches the full MetS condition (1111, at the right of the plot). This result suggests that microalbuminuria is related to MetS, as acknowledged by the WHO and consistent with previous reports relating hypertension and elevated proteinuria [40]. Yet, at worst (i.e., in condition 1111), only 10% of the samples show microalbuminuria, showing it to be only a secondary risk factor in the aetiology of MetS.

For the OSARTEN II and OBENUTIC cohorts, the estimated glomerular filtration rate (E-GFR) was determined from the available serum creatinine concentrations using the Chronic Kidney Disease Epidemiology Collaboration equation [41]. The values were sorted according to the G1-G5 scale (Additional file 1: Figure S5): most individuals (75%) fall in G1 category (normal or high GRF), 24.8% fall in G2 category (mildly decreased) and a residual percentage of individuals fall in G3a or G3b categories. None of the subjects have severely decreased GFR (G4) neither show kidney failure (G5). These results indicate that the observed metabolic changes are not biased by impaired renal function.

### Metabolic relationship between NAFLD and MetS

We have also investigated the putative relationship between MetS and NAFLD, the latter without discriminating between non-alcoholic fatty liver (NAFL) and NASH. Most of the RF defining MetS contribute to NAFLD progression and whether NAFLD is indeed the hepatic manifestation of NASH, as previously suggested [10], remains an open question. We analysed a cohort of 234 urines from patients with NAFLD, diagnosed and staged by liver biopsy, the reference method for the characterization of the disease [42]. Based on the WHO, EGIR and AACE criteria, samples were classified in two subcohorts: *NAFLD with MetS* and *NAFLD without MetS*. We then used our metabolic model to predict the probability of MetS for the two subcohorts. Figure 3B shows the pertaining probability distributions for the general population (apparently healthy, 0000), the *NAFLD without* or *with MetS* subcohorts and the MetS population (with unknown status about NAFLD). As expected, the *NAFLD without MetS* subcohort indeed shows a low probability for having MetS on average, with a very similar distribution to the general population (also without MetS), implying that the NAFLD associated metabotype differs from the one for MetS. This result is consistent with the lack of association between transaminase levels and MetS patients [24]. In contrast, the *NAFLD with MetS* subcohort shows a complex probability distribution, highlighting the fact that a simultaneous presence of NAFLD and MetS confounds the metabolic definition for the syndrome, suggesting a partial overlap of associated metabotypes in line with their common risk factors. Taken together, our results suggest that MetS and NAFLD may be comorbidities with distinct metabolic profiles, albeit with some overlapping features.

## Discussion

**Fig. 4:**
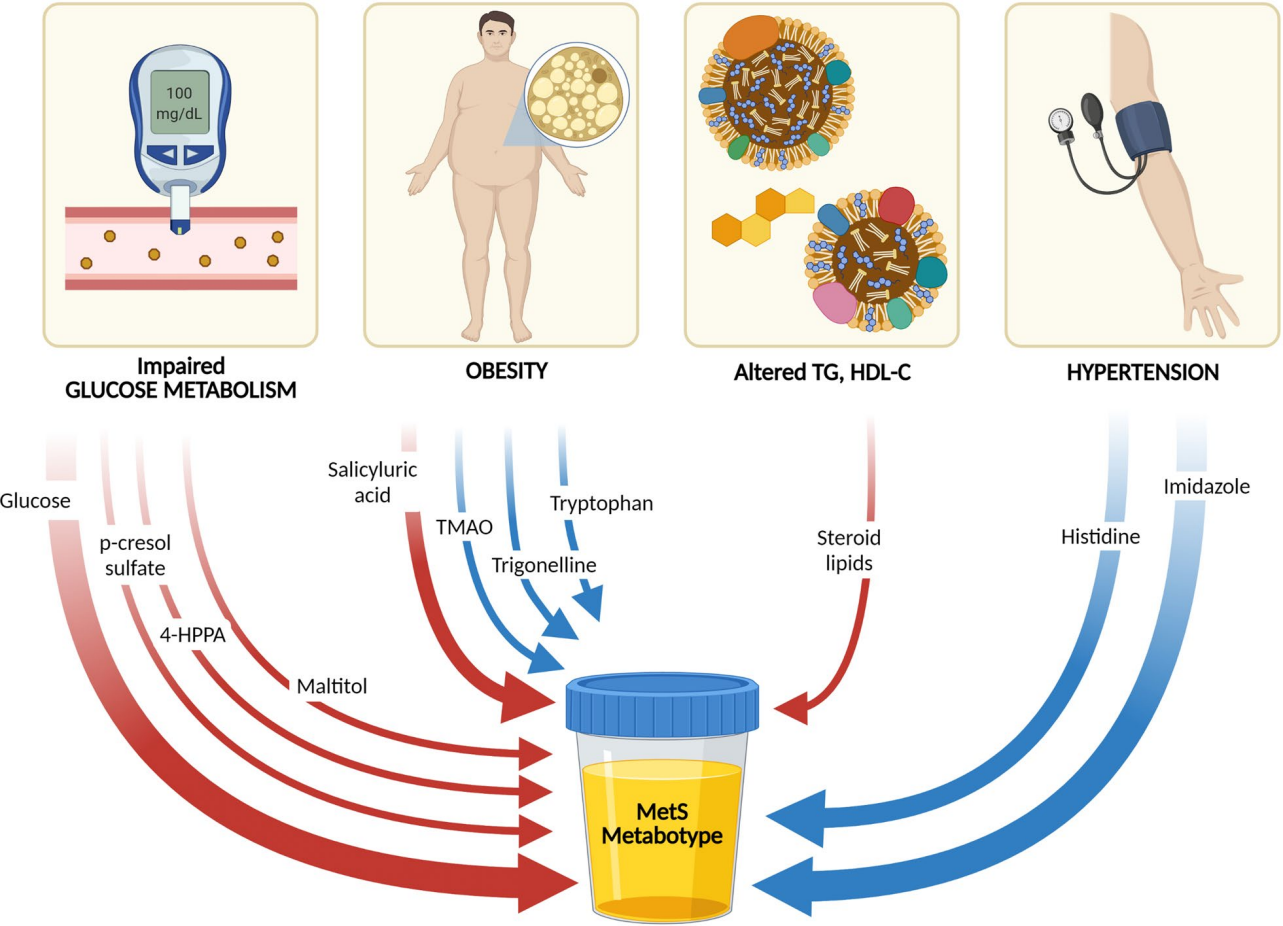
A molecular signature for MetS. All the risk factors that contribute to MetS have at least one metabolite in urine that is altered and contributes to the MetS metabotype. Such characteristic metabotype has been used to create a metabolic model to predict the probability of suffering MetS from the NMR analysis of a urine sample. Red and blue arrows correspond to up- and down-regulated metabolites in urine respectively. Created with BioRender.com

Our goal was to investigate the molecular signature of MetS in a large European cohort having a wide-range of MetS-related phenotypes. In here, we provide an unprecedented study using NMR spectroscopy and over a very large cohort of urine samples, specifically designed to populate all the possible intermediate conditions between healthy volunteers and MetS patients, the latter being characterized by the accumulation of RF and not biased by any specific definition of the syndrome. Remarkably, we always found a smooth but monotonic metabolic variation for a specific set of metabolites (Fig. 4 and Table 3), well-reflecting the progressive deterioration of the metabolism due to the accumulation of RF towards MetS. Any case, not all these factors contribute equally to MetS progression, providing a molecular signature of the syndrome, as highlighted by the risk factors enclosed in the orange ellipse in Fig. 1C. This molecular definition of MetS (conditions 1001, 1011, 1101 and 1111) may be of particular interest in the discrimination of conditions that are in the interface between healthy individuals and MetS patients.

Our molecular signature of MetS considers the problems with the metabolism of the glucose as a compulsory risk factor for MetS, in line with WHO definition. They include insulin resistance and are related with the pre-diabetic and diabetic state. These problems are well-reflected in our analysis by the high levels of glucose found. Other related metabolites include p-cresol sulfate, a uremic toxin that originates from tyrosine metabolism by intestinal microbes, also associated with insulin resistance [28], and 4-HPPA is also involved in this pathway. Finally, maltitol is a polyol used as a sugar derivative recommended in individuals at risk of T2D [43].

We also found hypertension a compulsory risk factor of MetS. Consistently, almost 80% of the patients affected by MetS present elevated blood pressure [44]. Lowered histidine and imidazole levels could be linked to an impairment in the concentration of the endogenous ligands of the imidazoline and α_2_-adrenogenic receptor, ultimately associated to hypertension episodes [29, 30]. In turn, dyslypidemia, directly reflected in the elevated levels of lipids in urine [24, 25] and obesity, monitored by abnormal levels of TMAO, trigonelline and salicyluric acid, contribute to MetS but they would not constitute essential risk factors according to our molecular signature of MetS.

The large number of samples in our study allowed to derive consistent metabolic models for discriminating MetS, adapted to the current existing definitions, and based only on a straightforward urine analysis by ^1^H NMR spectroscopy (with no need of adding characteristics from the individual). The target setting for our models was to compare our molecular definition of MetS with the current diagnostics for the syndrome (Table 1), adding a molecular dimension to its definition. All existing definitions, based on slightly differing sets of risk factors, agree well with our derived metabolic profile, with high AUROC values for discrimination ranging between 0.85 and 0.92, performing better than a previously reported model [45]. Here, the WHO, EGIR, and AACE definitions including diabetes as a compulsory risk factor for MetS condition agree best with our predictions from urine metabotyping, presumably owing to the important weight of urinary glucose in the metabolic model. Actually, the AUROC values would raise up to 0.86–0.92 if hyperglycemia is defined as glucose higher than 110 mg/dL (instead of 100 mg/dL, Additional file 1: Figure S6). Finally, our results also show a significant propensity for albuminuria in individuals with MetS, again in agreement with the WHO definition.

Finally, we also compared our urinary metabolic model for MetS, obtained from a vast and well-balanced sample cohort with the vast majority of them showing normal transaminase values, with an independent subcohort of NAFLD patients diagnosed by biopsy. While the results show a certain overlap of metabolic profiles between MetS and NAFLD, in agreement with their shared symptomatology, our MetS model can distinguish exclusive NAFLD condition without MetS comorbidity (Fig. 3B).

### Limitations of the study

The study is under the assumption that urine is sensitive to all the factors that contribute to MetS. Specifically, obesity and dyslipidemia induced lower changes, that could also be related to their intrinsic metabolic variability. Even though we found metabolites associated to all the risk factors in MetS, the inclusion of metabolomic information from other matrices (i. e. serum) is desirable.

## Conclusions

In summary, we have demonstrated that NMR-based metabolomics of urine samples can identify individuals with MetS condition. The relevant metabolites for discrimination are associated with all contributing risk factors, thus providing a holistic molecular signature for the metabolic syndrome. These results may improve clinical decision making and potentially guide early intervention in this important syndrome.

## Funding

Support was provided from The Department of Industry, Tourism and Trade of the Government of the Autonomous Community of the Basque Country (Elkartek BG2017 & BG2019); grant from Agencia Estatal de Investigación (Spain) RTI2018-101269-B-I00 and for the Severo Ochoa Excellence Accreditation (SEV-2016-0644). SL, JMM and OM are supported by National Institutes of Health (1U01 AA026817). This study was partially funded by the Generalitat Valenciana (Grant PROMETEO 17/2017 and APOSTD/2019/136); the Spanish Ministry of Health (Instituto de Salud Carlos III) and the Ministerio de Economía y Competitividad-Fondo Europeo de Desarrollo Regional (FEDER) (grants CIBER 06/03 and SAF2016–80532-R). EB, OM, QMA and JMM are supported by the LITMUS (Liver Investigation: Testing Marker Utility in Steatohepatitis) consortium funded by the Innovative Medicines Initiative (IMI2) Program of the European Union under Grant Agreement 777377–this Joint Undertaking receives support from the European Union’s Horizon 2020 research and innovation programme and EFPIA. QMA is supported by the Newcastle NIHR Biomedical Research Centre and is a European NAFLD Registry investigator.

## Supplementary Information

**Additional file 1. ** Additional Tables and Figures.

## Data Availability

The datasets used and analysed during the current study are available from the corresponding author on reasonable request.

## Abbreviations

MetS: Metabolic syndrome
NMR: Nuclear magnetic resonance
WHO: World Health Organization
IDF: The International Diabetes Federation
NCEP:ATP III: National Cholesterol Education Program-Third Adult Treatment Panel
EGIR: European Group for the Study of Insulin Resistance
AACE: American Association for Clinical Endocrinology
RF: Risk factors
IFG: Impaired fasting glucose
IGT: Impaired glucose tolerance
FG: Fasting plasma glucose
T2DM: Type 2 diabetes
WC: Waist circumference
WHR: Waist-hip ratio
BMI: Body mass index
TG: Triglycerides
HDL-C: HDL cholesterol
BP: Blood pressure
m: Male
f: Female
4-HPPA: 4-Hydroxyphenylpyruvic acid
TMAO: Trimethylamine *N*-oxide
E-GFR: Estimate glomerular filtration rate
