# Supplementary material for: A molecular signature for the metabolic syndrome by urine metabolomics
Source: Cardiovasc Diabetol. 2021 Jul 28;20:155. doi: 10.1186/s12933-021-01349-9 (PMC8320177; doi:10.1186/s12933-021-01349-9)
Supplement: Supplementary file 1 — Additional file 1. Additional Tables and Figures. [file 12933_2021_1349_MOESM1_ESM.pdf]

A molecular signature for the Metabolic Syndrome by urine metabolomics

Chiara Bruzzone[1,a], Rubén Gil-Redondo[1,a], Marisa Seco[2], Rocío Barragán[3,4], Laura de la Cruz[1], Claire Cannet[5], Hartmut Schäfer[5], Fang Fang[5], Tammo Diercks[1], Maider Bizkarguenaga[1], Beatriz González-Valle[1], Ana Laín[1], Arantza Sanz-Parra[1], Oscar Coltell[4,6], Ander López de Letona[7], Manfred Spraul[5], Shelly C Lu[8], Elisabetta Buguianesi[9], Nieves Embade[1], Quentin M Anstee[10,11], Dolores Corella[3,4], José M Mato[1], Oscar Millet[1,*]

[a]Both authors contributed equally

[1]CIC bioGUNE, BRTA, CIBERehd, Derio, Bizkaia, Spain.
[2]OSARTEN Kooperativa Elkartea, 20500 Arrasate-Mondragón, Spain.
[3]Department of Preventive Medicine and Public Health, School of Medicine, University of Valencia, 46010 Valencia, Spain.
[4]CIBER Fisiopatología de la Obesidad y Nutrición, Madrid, Spain
[5]Bruker Biospin GmbH, Silberstreifen, 76287 Rheinstetten, Germany.
[6]Department of Computer Languages and Systems, Universitat Jaume I, 12071 Castellón, Spain.
[7]Getxo Kirolak, Los Chopos Etorbidea, 56, 48992 Getxo, Bizkaia, Spain.
[8]Karsh Division of Gastroenterology and Hepatology, Cedars-Sinai Medical Center, Los Angeles, CA, USA.
[9]Gastroenterology Department, University of Turin, Turin, Italy.
[10]Translational & Clinical Research Institute, Faculty of Medical Sciences, Newcastle University, Newcastle-upon-Tyne, UK.
[11]Newcastle NIHR Biomedical Research Centre, Newcastle upon Tyne Hospitals NHS Trust, Newcastle upon Tyne, UK.

Table S1. General characteristics for the OSARTEN subcohort. Biochemical data have been extracted from a blood test and urine samples were collected at the same time.

	**[ALL valid]** ***N=9367***	**female** ***N=3432***	**male** ***N=5935***	**N**
Age (years)	43.04±9.16	44.06±8.73	42.45±9.35	9367
Weight (kg)	74.85±14.04	64.39±11.29	80.89±11.74	9367
Height (cm)	171.91±8.99	163.59±6.25	176.72±6.48	9367
BMI (kg/m²)	25.22±3.76	24.06±4.04	25.89±3.42	9367
Smoker	1877 (20.06%)	652 (19.03%)	1225 (20.66%)	9355
Alcohol consumption:				9345
Never	1325 (14.18%)	730 (21.33%)	595 (10.05%)	
Social drinker	6966 (74.54%)	2500 (73.04%)	4466 (75.41%)	
Only during meals	829 (8.87%)	171 (5.00%)	658 (11.11%)	
Daily intake of alcohol	225 (2.41%)	22 (0.64%)	203 (3.43%)	
Hypertension	985 (10.54%)	257 (7.50%)	728 (12.30%)	9343
Medicated for hypertension	86 (3.30%)	32 (3.04%)	54 (3.47%)	2610
Medicated for diabetes	70 (2.68%)	7 (0.66%)	63 (4.05%)	2609
Proteins detected in urine	126 (1.35%)	44 (1.29%)	82 (1.38%)	9359
ALT (U/L)	22.56±13.65	16.58±9.33	26.02±14.53	9367
Basophils (10^9/L)	0.04±0.02	0.04±0.02	0.04±0.02	9367
Cholesterol (mg/dL)	193.49±34.48	191.87±34.34	194.42±34.52	9367
HDL Cholesterol (mg/dL)	61.19±15.98	68.97±15.41	56.68±14.51	9367
LDL Cholesterol (mg/dL)	112.48±30.82	106.85±30.32	115.94±30.63	8878
Non-HDL Cholesterol (mg/dL)	132.30±35.97	122.90±34.05	137.74±35.93	9367
Mean corpuscular hemoglobin concentration (g/dL)	33.91±0.87	33.50±0.82	34.14±0.81	9367
Mean corpuscular volume (fL)	90.15±4.25	90.82±4.42	89.76±4.10	9367
Creatinine (mg/dL)	0.87±0.15	0.73±0.10	0.94±0.12	9367
Eosinophils (10^9/L)	0.23±0.16	0.21±0.16	0.24±0.17	9367
Erythrocytes (10^9/L)	4.83±0.41	4.50±0.31	5.03±0.34	9367
ESR (mm/h)	7.98±5.46	10.94±6.63	6.27±3.69	9367
GGT (U/L)	22.20±20.33	15.85±14.70	25.88±22.14	9366
Glucose (mg/dL)	85.68±12.00	84.19±10.08	86.54±12.90	9367
Hematocrit (%)	43.48±3.14	40.76±2.42	45.06±2.31	9367
Hemoglobin (g/dL)	14.75±1.21	13.65±0.91	15.38±0.86	9367
Leukocytes (10^9/L)	6.69±1.70	6.59±1.66	6.75±1.72	9367
Lymphocytes (10^9/L)	2.33±0.66	2.24±0.63	2.38±0.67	9367
Monocytes (10^9/L)	0.62±0.19	0.57±0.17	0.65±0.19	9367
Neutrophils (10^9/L)	3.48±1.26	3.54±1.29	3.45±1.25	9367
Platelets (10^9/L)	237.40±51.90	248.79±54.72	230.81±49.00	9367
Mean platelet volume (fL)	8.41±0.69	8.47±0.70	8.37±0.68	9366
Red cell distribution (U)	13.32±0.78	13.38±0.88	13.29±0.71	9367
Triglycerides (mg/dL)	96.72±63.06	78.63±37.34	107.18±71.92	9367
Urate (mg/dL)	5.10±1.26	4.13±0.92	5.66±1.07	9367

Table S2. General characteristics for the OBENUTIC subcohort. Biochemical data have been extracted from a blood test and urine samples were collected at the same time.

	**[ALL valid]** ***N=465***	**female** ***N=307***	**male** ***N=158***	**N**
Age (years)	46.14±13.67	46.42±12.94	45.61±15.01	465
BMI (kg/m²)	27.87±5.40	27.32±5.58	28.94±4.89	465
Waist (cm)	92.31±15.18	88.16±13.48	100.49±15.06	458
Smoker	93 (20.62%)	72 (23.92%)	21 (14.00%)	451
Systolic blood pressure (mmHg)	124.91±17.37	121.00±16.83	132.46±15.91	461
Diastolic blood pressure (mmHg)	78.62±10.69	76.94±9.71	81.89±11.74	461
Hypertension	76 (17.31%)	37 (12.76%)	39 (26.17%)	439
Medicated for hypertension	78 (17.33%)	35 (11.71%)	43 (28.48%)	450
Diabetes	21 (4.79%)	13 (4.48%)	8 (5.41%)	438
Medicated for diabetes	14 (3.12%)	8 (2.69%)	6 (3.97%)	448
Diagnosed cholesterol	132 (30.28%)	84 (29.17%)	48 (32.43%)	436
Medicated for cholesterol	67 (14.99%)	40 (13.47%)	27 (18.00%)	447
Any cardiovascular disease	15 (3.42%)	7 (2.41%)	8 (5.41%)	438
ALT (U/L)	25.29±18.61	20.85±11.96	33.86±25.11	463
AST (U/L)	25.85±11.04	23.33±7.15	30.73±14.95	461
Cholesterol (mg/dL)	212.93±40.11	216.62±40.51	205.77±38.45	465

	[ALL valid] *N=465*	female *N=307*	male *N=158*	N
HDL Cholesterol (mg/dL)	59.85±14.13	64.21±13.53	51.37±11.12	465
LDL Cholesterol (mg/dL)	138.50±32.52	138.86±33.00	137.79±31.67	464
Creatinine (mg/dL)	0.76±0.18	0.67±0.10	0.93±0.19	465
GGT (U/L)	30.74±32.46	27.65±33.59	36.73±29.32	461
Glucose (mg/dL)	94.66±19.07	92.68±16.35	98.50±23.05	465
Leukocytes (10^9/L)	6.43±2.51	6.47±2.89	6.33±1.49	443
Triglycerides (mg/dL)	109.42±58.70	102.63±52.88	122.64±66.87	463
Uric acid (mg/dL)	5.33±1.41	4.79±1.16	6.39±1.25	465

Table S3. General characteristics for the PREDIMED subcohort. Biochemical data have been extracted from a blood test and urine samples were collected at the same time.

	[ALL valid] *N=960*	female *N=612*	male *N=348*	N
Age group (years):				960
55-63	330 (34.38%)	213 (34.80%)	117 (33.62%)	
64-69	306 (31.87%)	191 (31.21%)	115 (33.05%)	
70-80	324 (33.75%)	208 (33.99%)	116 (33.33%)	
Diabetes	490 (51.04%)	279 (45.59%)	211 (60.63%)	960
Obesity	492 (51.25%)	338 (55.23%)	154 (44.25%)	960
Dyslipidemia	303 (31.56%)	177 (28.92%)	126 (36.21%)	960
Hypertension	798 (83.12%)	524 (85.62%)	274 (78.74%)	960

Table S4. General characteristics for the KIROLGETXO subcohort. Data have been collected from questionnaries.

	[ALL valid] *N=101*	female *N=83*	male *N=18*	N
Age (years)	71.14±5.46	70.87±5.49	72.39±5.29	101
Weight (kg)	66.81±12.22	65.08±11.73	74.78±11.51	101
Height (cm)	161.12±7.35	159.65±6.66	167.89±6.68	101
BMI (kg/m²)	25.67±3.95	25.50±4.04	26.48±3.47	101
Ethnic group:				101
caucasian	100 (99.01%)	82 (98.80%)	18 (100.00%)	
hispanic	1 (0.99%)	1 (1.20%)	0 (0.00%)	
Smoker	4 (3.96%)	3 (3.61%)	1 (5.56%)	101
Alcohol consumption:				101
Never	26 (25.74%)	23 (27.71%)	3 (16.67%)	
Social drinker	50 (49.50%)	46 (55.42%)	4 (22.22%)	
Only during meals	16 (15.84%)	10 (12.05%)	6 (33.33%)	
Daily intake of alcohol	9 (8.91%)	4 (4.82%)	5 (27.78%)	
Physical exercise: Several days per week	101 (100.00%)	83 (100.00%)	18 (100.00%)	101
Diabetes	5 (4.95%)	2 (2.41%)	3 (16.67%)	101
Medicated for diabetes	7 (7.00%)	4 (4.82%)	3 (17.65%)	100
Hypercholesterolemia	38 (37.62%)	29 (34.94%)	9 (50.00%)	101
Medicated for hypercholesterolemia	25 (25.00%)	19 (22.89%)	6 (35.29%)	100
Hypertension	32 (32.00%)	25 (30.12%)	7 (41.18%)	100
Medicated for hypertension	29 (29.29%)	24 (28.92%)	5 (31.25%)	99
Any cardiovascular disease	13 (13.00%)	9 (10.98%)	4 (22.22%)	100
Medicated for cardiovascular disease	9 (9.57%)	5 (6.49%)	4 (23.53%)	94

Table S5. General characteristics for the NAFLD subcohort. Biochemical data have been extracted from a blood test and urine samples were collected at the same time.

	**[ALL valid]** ***N=234***	**female** ***N=89***	**male** ***N=145***	**N**
Age (years)	54.49±11.92	58.04±11.38	52.30±11.75	234
BMI (kg/m²)	32.26±7.63	32.17±7.45	32.32±7.76	233
Waist (cm)	110.36±14.03	108.65±14.76	111.30±13.58	175
Ethnic group:				231
African	1 (0.43%)	1 (1.14%)	0 (0.00%)	
Arab	3 (1.30%)	1 (1.14%)	2 (1.40%)	
Bangladeshi	1 (0.43%)	0 (0.00%)	1 (0.70%)	
Caribbean	1 (0.43%)	1 (1.14%)	0 (0.00%)	
Indian	4 (1.73%)	1 (1.14%)	3 (2.10%)	
Other Asian background	2 (0.87%)	1 (1.14%)	1 (0.70%)	
Other Black/African/Caribbean background	3 (1.30%)	0 (0.00%)	3 (2.10%)	
Pakistani	2 (0.87%)	0 (0.00%)	2 (1.40%)	
White	214 (92.64%)	83 (94.32%)	131 (91.61%)	
Diabetes	124 (52.99%)	59 (66.29%)	65 (44.83%)	234
Medicated for diabetes	96 (41.20%)	41 (46.07%)	55 (38.19%)	233
Medicated for hypercholesterolemia	108 (46.15%)	47 (52.81%)	61 (42.07%)	234
Medicated for hypertension	123 (52.56%)	54 (60.67%)	69 (47.59%)	234
NAFLD Activity Score (NAS)	4.18±1.53	4.38±1.58	4.06±1.49	233
NAS - Steatosis:				234
1	89 (38.03%)	31 (34.83%)	58 (40.00%)	
2	83 (35.47%)	31 (34.83%)	52 (35.86%)	
3	62 (26.50%)	27 (30.34%)	35 (24.14%)	
NAS - Lobular inflammation:				234
0	28 (11.97%)	12 (13.48%)	16 (11.03%)	
1	139 (59.40%)	51 (57.30%)	88 (60.69%)	
2	64 (27.35%)	24 (26.97%)	40 (27.59%)	
3	3 (1.28%)	2 (2.25%)	1 (0.69%)	
NAS - Hepatocellular ballooning:				233
0	42 (18.03%)	13 (14.77%)	29 (20.00%)	
1	125 (53.65%)	43 (48.86%)	82 (56.55%)	
2	66 (28.33%)	32 (36.36%)	34 (23.45%)	
Fibrosis stage:				234
0	40 (17.09%)	14 (15.73%)	26 (17.93%)	
1	23 (9.83%)	6 (6.74%)	17 (11.72%)	
1a	18 (7.69%)	10 (11.24%)	8 (5.52%)	
1b	17 (7.26%)	5 (5.62%)	12 (8.28%)	
1c	16 (6.84%)	4 (4.49%)	12 (8.28%)	
2	31 (13.25%)	7 (7.87%)	24 (16.55%)	
3	60 (25.64%)	26 (29.21%)	34 (23.45%)	
4	29 (12.39%)	17 (19.10%)	12 (8.28%)	
NAFLD diagnosis:				234
Steatosis	86 (36.75%)	31 (34.83%)	55 (37.93%)	
NASH	148 (63.25%)	58 (65.17%)	90 (62.07%)	
Albumin (g/L)	44.01±3.65	42.78±3.44	44.77±3.58	204
ALT (U/L)	57.02±37.22	51.69±35.03	60.28±38.26	229
AST (U/L)	40.62±24.72	41.86±28.31	39.87±22.32	229
Glucose (mg/dL)	120.66±44.00	132.41±50.87	114.04±38.29	172
Triglycerides (mg/dL)	190.88±293.61	243.42±455.02	158.37±98.11	225

Table S6. Number of samples allocated to each condition under study.

	[ALL valid] *N=10792*	female *N=4351*	male *N=6441*	N
MetS condition:				10792
0000	6925 (64.17%)	2935 (67.46%)	3990 (61.95%)	
0001	692 (6.41%)	276 (6.34%)	416 (6.46%)	
0010	733 (6.79%)	120 (2.76%)	613 (9.52%)	
0011	170 (1.58%)	53 (1.22%)	117 (1.82%)	
0100	504 (4.67%)	232 (5.33%)	272 (4.22%)	
0101	310 (2.87%)	169 (3.88%)	141 (2.19%)	
0110	170 (1.58%)	37 (0.85%)	133 (2.06%)	
0111	148 (1.37%)	62 (1.42%)	86 (1.34%)	
1000	282 (2.61%)	89 (2.05%)	193 (3.00%)	
1001	188 (1.74%)	83 (1.91%)	105 (1.63%)	
1010	84 (0.78%)	18 (0.41%)	66 (1.02%)	
1011	90 (0.83%)	32 (0.74%)	58 (0.90%)	
1100	92 (0.85%)	44 (1.01%)	48 (0.75%)	
1101	202 (1.87%)	111 (2.55%)	91 (1.41%)	
1110	62 (0.57%)	17 (0.39%)	45 (0.70%)	
1111	140 (1.30%)	73 (1.68%)	67 (1.04%)	

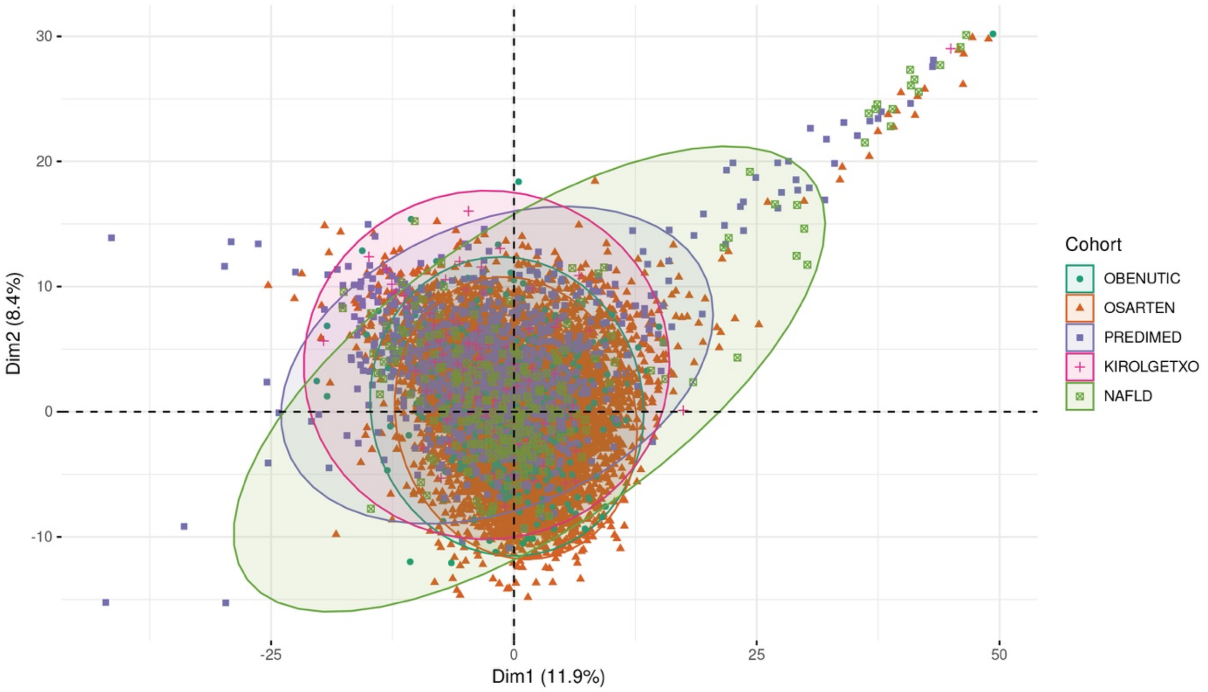

Figure S1. PCA analysis of the different subcohorts under consideration in the present study. The overlap in the PC space validate their integration for the investigation of MetS.

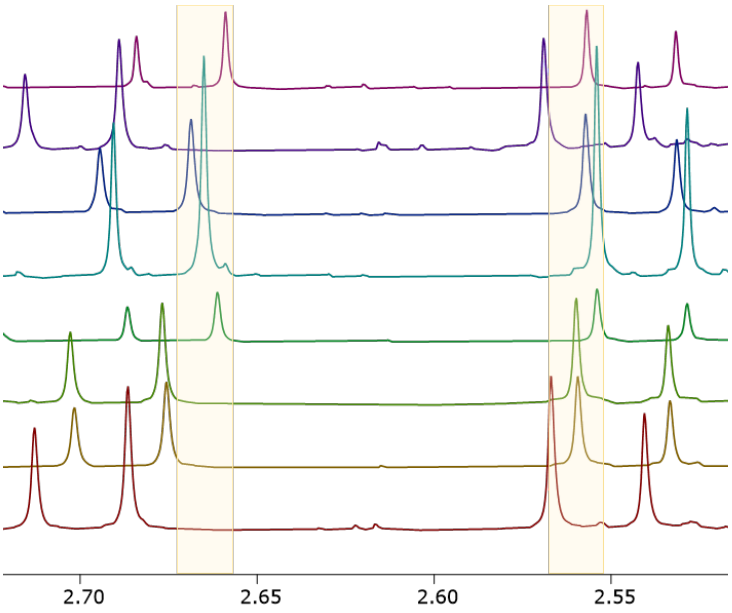

Figure S2. Representative stack plot of urine spectra where the citrate signals are highlighted (yellow boxes). Please notice the strong chemical shift dependence of the citrate signal with the matrix conditions (i. e. pH and osmolarity).

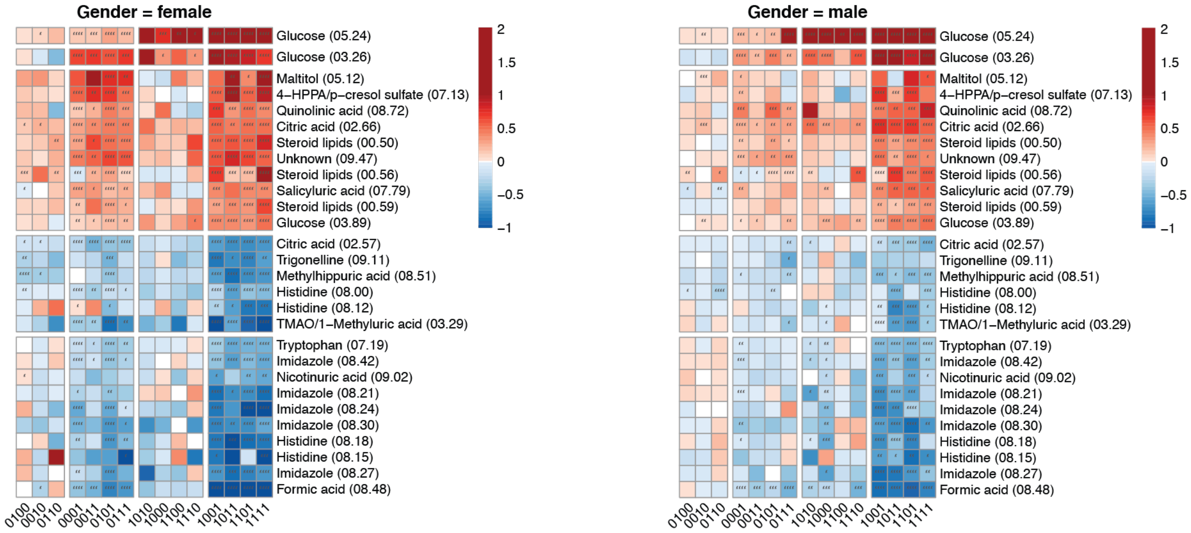

Figure S3. Heatmaps for the different conditions as compared to the asymptomatic condition (0000), segregated by gender (women left, men right, as indicated). The conditions (in the abscise axis) and the bins/metabolites (in the ordinate axis) have been sorted according to original cluster analysis. The relevant bins that contributed to the heatmap have been assigned to the corresponding metabolite, as indicated. The fold change is colour-coded according to the bar legend. For each condition, the statistical significance of the variation with respect to asymptomatic individuals is determined by the p-value, shown inside the squares. 4-HPPA: 4-hydroxyphenylpyruvic acid; TMAO: trimethylamine N-oxide.

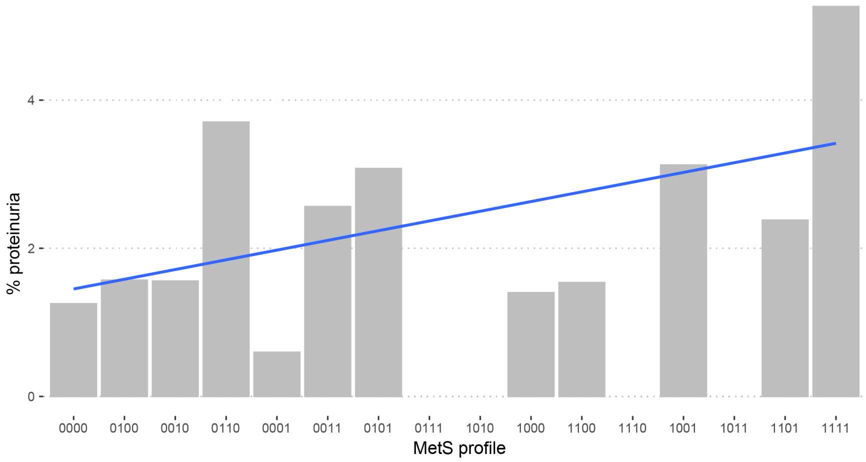

Figure S4. Bar plot with the percentage of samples with albuminuria (albumin in urine > 10 mg/dL) as a function of the condition, for the OSARTEN cohort. Conditions are sorted according to heatmap order (Figure 1B). The line shows the linear regression to the values assuming equidistance in the conditions towards MetS.

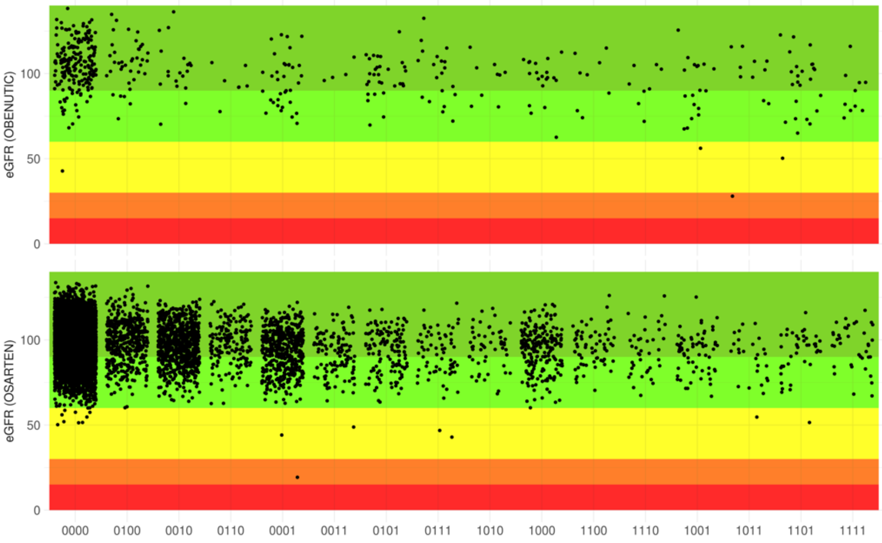

Figure S5. Distribution of E-GFR values for the OSARTEN and OBENUTIC cohorts, as a function of the MetS conditions. The E-GFR values are colour coded according to the categories (G1 to G5): G1, normal or high E-GFR (dark green); G2, mildly decreased E-GFR (green); G3, Moderate to severely decreased E-GFR (yellow); G4, severely decreased E-GFR (orange); G5, kidney failure (red).

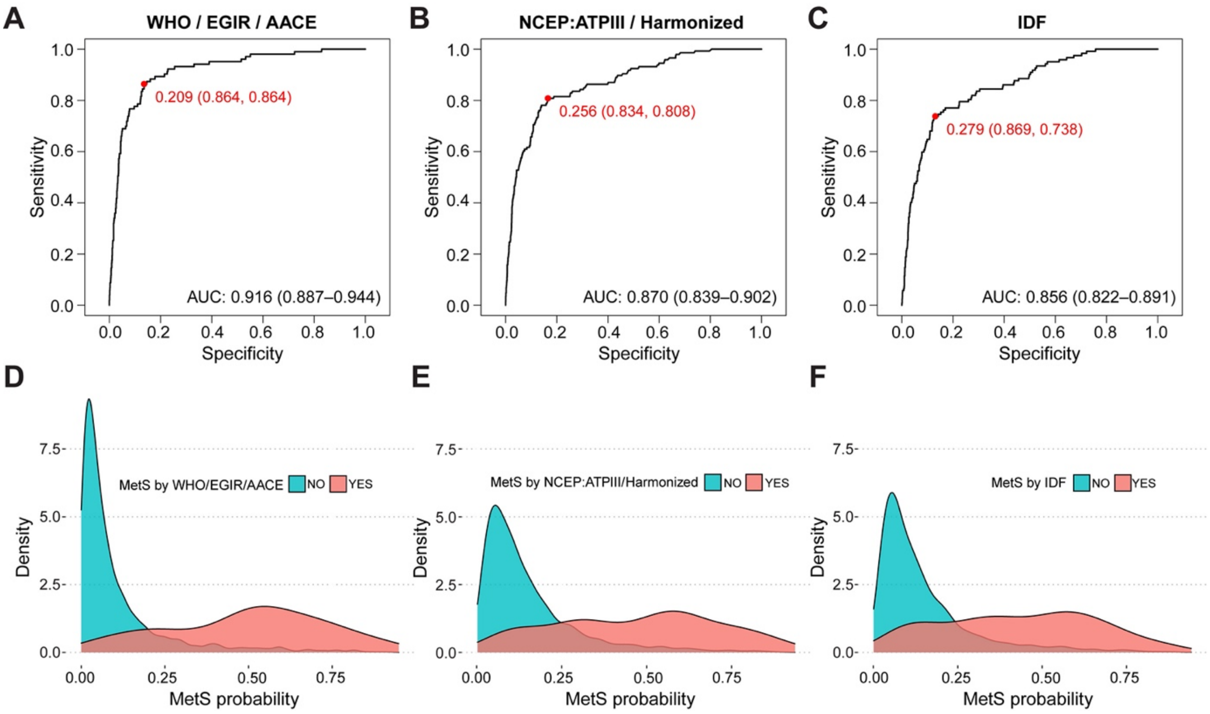

Figure S6. Probability distribution of the MetS models with a glucose cutoff value of 110 mg/dL. A-C) Receiving Operating Characteristic (ROC) curves for the three definitions under consideration: WHO, EGIR and AACE (A), NCEP:ATPIII and Harmonized (B), and IDF (C). D-F) smoothed histograms (kernel density based) showing the probability distributions of the MetS model applied to the full cohort for the three definitions under consideration: WHO, EGIR, and AACE (D), NCEP:ATPIII and Harmonized (E), and IDF (F). Red and green colours indicate that the sample has/doesn't have MetS according to the given definition, as indicated.
